# Frail Hypotheses in Evolutionary Biology

**DOI:** 10.1371/journal.pgen.1001067

**Published:** 2010-09-16

**Authors:** Jacques Ninio

**Affiliations:** Laboratoire de Physique Statistique de l'Ecole Normale Supérieure, UMR 8550 of the CNRS, UPMC Université Paris 06 and Université Paris Diderot, Paris, France; Université Paris Descartes, INSERM U571, France

In the last decades, under the headings of “mutation strategies,” “evolvability,” or “soft inheritance,” many ideas have been advanced on mechanisms assumed to promote innovative evolution beyond what one may anticipate from the classical model of random mutation and selection. Many population geneticists find these ideas superficially seducing but mathematically unfounded. While agreeing with the need to critically evaluate such proposals in the light of population genetics, I will argue that population geneticists are not immune to criticism. For instance, the “infinite site model” introduced by Kimura makes the unrealistic assumption that any neutral mutation arises only once during a neutral fixation episode, which leads, I propose, to an underestimation of the neutral fixation rates in large populations. Critical parameters such as mutation and recombination rates, effective population sizes or beneficial/deleterious mutation ratios are assigned convenient values, which may seem ad hoc to people outside the field. The lack of concern for the subtleties of genetic mechanisms is also criticized. Phenomena such as compensatory mutations, recurrent mutations, hot spots, and polymorphism, which population geneticists treat in the mathematical context of neutral versus selective fixations, can instead be interpreted in terms of genetic mechanisms for producing complex mutational events. Finally, single nucleotide substitutions are often treated as the quasi-exclusive source of variations, yet they cannot help much once the genes are optimized with respect to these substitutions. I suggest that population geneticists should invest more effort in refining the numerical values of the critical parameters used in their models. They should take into account the recent proposals on how mutations arise. They should also pay more attention to phenotypic variations, and develop criteria to discriminate between proposed evolutionary mechanisms that can actually work, and others that cannot.

## Smart Evolutionary Devices?

For over a century, inventing an adaptive story for each particular trait in a species has been a major pastime of evolutionary biologists [1], [2]. This activity lost some of its appeal under the strokes of neutralist theories, according to which most of the nucleotide variations in DNA sequences of higher organisms are either selectively neutral [3] or even slightly deleterious [4]. The new trend is to propose smart evolutionary strategies based on each newly discovered form of genetic or phenotypic plasticity.

There are subtle ways of producing point mutations [5], and many forms of “natural genetic engineering” including transposition, reverse transcription, exon shuffling, combinatorial recombination, RNA editing, horizontal gene transfer [6]–[8]—the list is still expanding [9]. There are also “soft” inheritable variations, more easily reversed than point mutations [10]–[12]. Among these, DNA methylation and chromatin modifications have been proposed as agents in smart evolutionary mechanisms [13]–[14]. A classical theme underlying these proposals is that all forms of genetic and phenotypic variability are under genetic control, so when a beneficial mutation is fixed by natural selection, the gene controlling the production of such mutations is driven to fixation by hitchhiking.

In a remarkable article, Michael Lynch [15] offered a case by case refutation of recent proposals on smart evolution, asking with great clarity, “Have evolutionary biologists developed a giant blind spot; are scientists from outside the field reinventing a lot of bad wheels; or both?”

I do worry about bad wheels, remembering from thermodynamics that all proposals for perpetual motion machines turned out to be flawed. However, I also know that contrary to the formal proofs of yore, objects heavier than air can in fact fly. I will therefore question some current assumptions in population genetics and then present some subtleties of the mutation processes not yet taken into account in evolutionary biology. Finally, I will discuss the soft variation issue and issues in innovative evolution.

## On Mutation and Fixation Rates

The neutral theory of molecular evolution [3] plays a central role in population genetics. Unfairly attacked as “anti-Darwinian” in the beginning, it now enjoys a status comparable to that of ideal gases in physics [16]. It leads to miraculously simple relations on fixation probabilities, number of generations to fixation, and heterozygosity level per locus. Once it is decided, in molecular evolution studies, that variations at some sites are neutral (for instance, synonymous codon substitutions, or mutations in junk DNA), the nature and strength of selection are deduced from the rates of variation at other sites.

There is in the neutral theory a simplifying mathematical assumption called “the infinite site model,” according to which any given mutation “has all the time it needs” to be either fixed or eliminated, before a second mutation arises at the same locus in the population. This assumption is unrealistic in most practical cases. Consider a population of size N and the classical neutral fixation time of 4N generations, encompassing 4N^2^ individuals. Take, for instance, an animal population of size 10^5^ and a mutation rate of 10^−8^ per site per generation, as in humans [17]. Then any particular mutation would occur well over a hundred times during a 4N generations span.

According to one line of reasoning, when a mutation is spreading, the occurrence of other similar mutations would have little impact, because only about 1/N of the new mutations would be expected to survive drift. However, there is a conceptual difficulty with variations that propagate from multiple sources. If you consider the tree generated from the mutational event A when the mutant population has reached a size m, and you introduce a similar mutational event B, this event would change the fixation probability of A by roughly (m+1)/m, which is in most cases negligible. But considered from the side of B, the tree generated from B has a substantially increased fixation probability. It merely needs to expand into a non-mutant population of initial effective size N-m-1, instead of N-1.

On the whole, I expect that after correction for back mutation and tree merging, neutral fixation times will turn out to be significantly shorter than predicted from the infinite site assumption. Corrections for multiple occurrences of mutations should be large in the case of neutral mutations drifting in large populations, and smaller in the case of selected mutations, because the shorter fixation times of the latter reduces the probability of multiple occurrences. At a deeper conceptual level, the infinite site model creates a blind spot, because it distracts us from thinking about classes of evolutionary events that occur repeatedly, perhaps through different channels.

This analysis will leave many evolutionary biologists unsatisfied. According to one Reviewer, for instance, “The major benefit of population genetics is that it allows quantitative effects to be measured with either pure mathematics or with simulations. As the hypotheses are clearly stated, their range of validity can be challenged! So criticism can not just be based on hand waving, as it is the case here for the infinite site.”

In a discussion of the current limitations of population genetics, Wakeley writes “It is problematic when conclusions drawn from a special case of a general model become normative statements carried over to other situations” [18]. Too often, I suggest, population geneticists succumb to the power and elegance of their mathematical treatments, but pay too little attention to the actual values of the parameters used in their models. As emphasized by many authors, the “effective population size” is treated as an adjustable parameter, not an experimental one.

Theoretical treatments of mutation rate optimality require precise data on the partitioning between neutral, beneficial, and deleterious mutations, but mathematical sophistication is not often matched by attention to the parameter's numerical values. In recent treatments [19], the deleterious/beneficial mutation ratio is assumed to be as high as four to five orders of magnitude, implying that *E. coli*'s genome is fully optimized with respect to single nucleotide substitutions. The deleterious mutation rate would be higher than 2×10^−4^ per genome replication, or about one tenth the mutation rate [20]. On the other hand, “the proportion of mutations that are beneficial is roughly one in a million” [21].

Intuitively, the postulated overwhelming excess of deleterious mutations cannot be true at the early stages of the evolution of a new function. From studies on acquisition of a promiscuous new function Bershtein and Tawfik [22] calculated a deleterious/beneficial mutation ratio of 360. The ratio might even be lower if the new function evolves from a random [23] or a repetitive [24] sequence. Gene optimization cannot be extensive in higher organisms. This is true to the point that many population geneticists worry about the mutation load, particularly in the human species [25], [26]. “Why aren't we extinct?” Crow asks [26], commenting on Keightley and Eyre-Walker [27].Thus, human populations must contain large numbers of genes that can be improved by single nucleotide changes.

Are mutation rates optimal? Sturtevant [28] reasoned on *Drosophila* and assumed a wide predominance of unfavourable mutations. He reasoned that for every favourable mutation with even a 1/1000 selective advantage “the preservation of which will tend to increase the number of genes in the population that raises the mutation rate, there are hundreds of unfavourable mutations that will tend to lower it.” On these grounds, the mutation rate should tend to zero, if it were not for the fact that “mutations are accidents, and accidents will happen.” Both upward and downward trends in mutation rates have been observed.

In laboratory work on bacterial growth under sustained selective pressures, mutator bacteria are selected [29]–[31]. If the mutator state is due to the loss of a key component of the mismatch repair (MMR) system, clonal reproduction of these bacteria should lead to extinction. Salvation occurs in nature because the missing MMR components are readily acquired through genetic exchanges between bacteria [32].

Noting that in general, “the most common class of mutations is to temperature sensitivity,” John Drake reasoned that the thermostability requirement would put severe constraints on protein sequences in thermophiles, implying that the proportion of deleterious mutations would be rather high in these organisms, thus favouring a low mutation rate [33]. Indeed, the mutation rate in two thermophiles—an archeon and a bacterium—appears to be five times lower than in non-thermophilic bacteria [33].

Still, I find that the standard mutation rate in bacteria (3×10^−3^ per genome replication) is amazingly low. In my opinion, the low value is used to maintain close to a functional state cryptic genes that are sporadically useful—a proposal which deserves being validated or refuted by population genetics. An alternative explanation is that higher mutation rates (in the 10^−1^ per genome replication range) would not be compatible with the maintenance of the housekeeping machinery, and would ultimately lead to error catastrophe.

## The Multiple Origins of Point Mutations

I now discuss some subtle aspects of mutation rates heterogeneity that, I propose, have deep implications on molecular evolution [34]–[36]. A first insight is that mutation rates heterogeneities make double mutation events far more frequent than predicted by the single mutation frequencies [34]. A second insight is that even a “nonmutagenic repair system” is error-prone, so while repair systems remove a large number of simple mistakes, they can introduce a small number of complex mutations when they resynthesize DNA [35], [36].

### Mutations by Legitimate Repair

It now seems that all repair systems have their errors. Mismatch repair involves the degradation of a 300- to 2,000-nucleotide DNA patch, followed by its re-synthesis. If ten thousand mismatches are detected and subject to correction, and if one hundred errors are made in the correction process, the MMR system would have reduced the errors by a hundred-fold factor. In this respect, it is nonmutagenic. But double mutations may have been occasionally introduced in some repair patches, at a significantly higher frequency than in the other sections of the genome [35]. I further speculate here that a similar strategy may be applied before “legal” repair. A standard DNA polymerase, having made a mistake and left it uncorrected, may be hindered in its progression by the DNA defect about 10 nucleotides later. Then, it might switch to a processive exonuclease mode and resume synthesis in error-prone mode—a behaviour previously described for *E. coli* Pol. I [37]. The existence of multiple working modes could perhaps explain strange observations on multiple errors in in vitro replication [38].

### Mutations by Overzealous Repair

Stretches of strictly complementary DNA, perhaps 10- to 12-nucleotides long, might act as preferential targets for the MMR system. They would act as though they contained “illusory mismatches” [36]. Such sequences would behave as strange mutational hot spots. DNA re-synthesis of these patches during gratuitous repair would generate, with a small probability, re-synthesis errors in their vicinity. But since repair will usually regenerate exactly the initial illusory mismatch, the small sequence is likely to be again and again the target of attacks by the MMR system, becoming a mutation hot spot until it is destroyed due to erroneous repair [36]. Recent studies of local inhomogeneities in mutation rates have in fact revealed a new kind of hot spots, having, I believe, the properties expected from the illusory mismatch principle [39].

Note that overzealous repair is known to produce real mutations in the case of base-excision repair [40], [41], and that somatic generation of antibody diversity follows a similar principle. A local DNA sequence is recognized, an adenine in this sequence is chemically modified, then a DNA repair system detects the anomaly, degrades a DNA patch, and re-synthesizes it again and again in an error-prone mode [42]–[44].

### Phenotypic Variations and Transient Mutators

Mutation bursts can be produced as a result of phenotypic accidents or phenotypic states that deviate from the regular state. Thus, an error-prone DNA polymerase may be synthesized as a result of translation or transcription errors. The MMR may be lacking an essential component due to unequal partitioning of its molecules at cell division. The cells in which these phenotypic accidents occur may produce mutations at a significantly higher frequency than wild-type, but their mutator state is transient and disappears after one or a few generations. Simple calculations suggest that in an *E. coli* population growing without selective pressures, such “transient mutators” [34] represent about 5×10^−4^ of the whole population. In the non-selective case, they would be about 50 times more numerous than the authentic genotypic mutators. Calculations on the incidence of one type of error on other types of errors have been pursued systematically for *E. coli* [45] and extended to higher organisms [46].

There was a widespread enthusiasm in the 1990s about “directed mutation mechanisms,” according to which bacterial genetic systems are organized in such a way that mutations are produced preferentially where they are needed [47], [48]. Such proposals were based on laboratory experiments in which a gene was inactivated then restored by spontaneous mutation. Detailed analyses on the recovery pathways are generating vigorous debates. Several but not all [47]–[50] authors favour a scheme in which the selective conditions generate stress, which triggers more or less directly error-prone repair systems, which produce mutation bursts.

In both the cases of transient mutators, which apply to non-selective conditions, and stress-induced mutations, there would be inhomogeneities in the mutation rates, producing double mutation events at a significantly higher frequency than expected from the single mutation frequency. Massive DNA sequencing suggests this is the case, not only in bacteria, but at all levels of life [38], and some genetic observations point in the same direction [51]. Clearly, many population genetics treatments (e.g., about compensatory mutations, or about linkage disequilibrium) should take into account, if not the transient mutator concept, at least the experimental facts about multiple mutations [38].

### On Some Subtleties of Recombination and Gene Conversion

Recombination, in population genetics, is presented as a shuffling mechanism, which generates new allele combinations on a chromosome. Recombination events as defined now may or may not involve crossing over—a typical ratio could be five non-crossovers for each crossover event [52]. Therefore, the shuffling role is not prominent. Each recombination event involves the degradation of a 300- to 2,000-nucleotides-long patch of DNA, as in MMR, and re-synthesis of the patch by copying a DNA strand from the homologous gene on the other chromosome, amounting to a gene conversion. If such a phenomenon occurs early in the germ line, and the strands were initially heterozygous, there would be a reduction of polymorphism transmitted to the next generation. From this perspective, recombination rather than creating diversity, has a streamlining effect. Next, recombinational DNA re-synthesis being made in error-prone mode [53], [54] mutations are introduced, so a recombination hot spot becomes a mutation hot spot - now a well accepted idea [55], [56].

Assume that recombination occurs preferentially close to DNA positions in which there is some divergence between two alleles. For instance, there could be a mechanism of sequence comparison between the two allelic sequences, generating double-strand breaks preferentially where heteroduplexes are detected. To me, this view seems consistent with genetic findings [57]–[59]. Assuming that a moderate heterozygosity in the sequences of the two alleles of a gene favour gene conversion, we would have a mechanism for enhancing the mutation rate in polymorphic regions. This comes naturally in relation to molecular drive [60] in repeated sequences, microsatellites in particular [61], but I deal here essentially with point mutations. Instead of conceiving polymorphism as a passive reflection of mutation pressure, polymorphism would be an active promoter of mutations through recombination hot spots, until a sequence is created which confers a substantial selective advantage, then is rapidly fixed [35], [62]. Mutation hot spots would be, by nature, transient [56]. A main insight in this analysis is the existence of classes of mutation which are boosted by heterozygosity (e.g., [63] and other references in [62]). An observation which could make sense, in such a scheme, and be relevant to human pathologies, is that of independent mutations in a same gene, arising in small populations [64]–[66].

## Phenotypic Versatility and Innovative Evolution

Once genes are optimized with respect to single nucleotide substitutions, further optimization requires more drastic genetic variations or qualitatively different mechanisms of variations. There are many forms of post-transcriptional modifications in RNA molecules and many classes of post-translational modifications in proteins, including phosphorylation and dephosphorylation systems in regulation networks, and chromatin methylations. The modifying enzymes act in a diffuse manner on many targets, the modifications are not always complete, generating a heterogeneity that varies with cell type and cell age. Molecular biologists used to consider the modifications one at a time. Presumably, the real producer of selective advantages is the balance of the modifications of a given kind over all the targets. In higher organisms, the complexity of regulatory networks is bewildering, but deceptive. You can erect a statue over a heap of stone, after adding cement to the heap. Afterwards, each stone may look important, and each contact point between a stone and its neighbours may look crucial, yet the stones initially formed an unstructured heap.

Microbial populations encounter a variety of conditions and possibly go through periods of reduced translation accuracy. In this case, the product of a gene is the standard translation sequence plus a large number of variants. Then, in a sense, the organism explores the sequence space around each coding gene, and fitness is related to the coding gene neighbourhood [67]–[69]. This and other arguments suggest that the sequence space is rather smooth around coding genes in micro-organisms, this being an evolved property [70], [71], but it cannot be so smooth in higher organisms [46]. Note that according to in silico studies, natural selection would fail to optimize mutation rates on rugged fitness landscapes [72]. At least in bacteria, highly selected genes are somewhat buffered, and they may contain information about “underground” activities that are useful in rare circumstances [73], or about the catalytic properties of single nucleotide substitutions [74]. Metabolic networks are also believed to be buffered against simple mutations. Increasing the efficiency of any particular component may have a negligible influence on the global efficiency of the network, a necessary [75] or evolved [76] property.

Another aspect of variability to consider is the capacity to deal with a range of environments. An organism acts as though it has several alternative genetic programs which can be unfolded, depending upon the circumstances [77], [78]. According to Lindquist, Rutherford, and other authors, the Hsp90 chaperone may play the role of an “evolutionary capacitor” [79], [80]. It would buffer the effect of certain mutations, thus reducing the mutational burden without reducing genetic polymorphism. Symmetrically, there would be a release of genetic variation when Hsp90 is repressed under stress conditions, thereby revealing normally silent polymorphism.

The immune system can design novel antibodies, in response to compounds never encountered before, and maintain a memory of the most successful responses. It is believed that the maturation of the nervous system is also subject to custom-fit adaptations. How does regulation in higher organisms cope with the genetic novelty of each newborn individual? Are there mechanisms for self-tuning? The metabolic networks are perhaps subject to custom-fit fine-tuning, through phosphorylation-dephosphorylation mechanisms [81], but this has not yet been proved.

A most ingenious link between phenotypic and genotypic variations was made very early by James Mark Baldwin [82]. His model still makes perfect sense when transposed into the language of molecular genetics. Imagine a genetically homogeneous population under selective pressure. Since the phenotypic variability associated with the standard genome may be high, some members of the population may have a deviant phenotype well adapted to the selective pressure. These will survive, and perpetuate the species with its standard phenotypic variability, until a mutation arises which produces, genotypically, the helpful phenotype as a more central phenotype. Hence, the genotype somehow copies the phenotype, and this event is named a phenocopy. In his youth, Piaget made observations on genotypic and phenotypic variations in plants as a function of altitude, which he interpreted in terms of a Baldwin effect, as discussed later in his book on vital adaptation [83].

Transcriptional infidelity may promote, under special conditions, inheritable phenotypic changes [84]. Note, however, that the Baldwin effect is not about the individual inheritability of a phenotype. It is about phenotypic variability that is statistically reproducible at the population level.

The extent of phenotypic variations depends on population size. For instance, in very large populations, there may be double transcription errors in a gene, generating proteins with quadruple changes, creating phenotypes far removed from the standard genotype [38], [46]. Large populations may escape from extinction under harsh conditions, with greater probability than predicted classically from their reduced waiting time for beneficial mutations. Phenotypic diversity goes to an extreme in the immune system, due to the mechanisms for the generation of antibody diversity. Therefore this is a domain in which evolution may be accelerated by a Baldwin effect.

While we need to consider the many phenotypes arising from a single genotype in the first phase of the Baldwin effect, we must remain aware of the possibility that many different mutations, in many different genes may generate the beneficial phenotype in the second phase. Actually, a recurrent observation in experimental evolution is that there are multiple genetic ways of producing a same effect, e.g. [85].

## Conclusion

In conclusion, I return to Michael Lynch's challenging questions about blind spots and bad wheels in evolutionary biology which motivated this review [15]. Concerning blind spots I have pointed out some limitations of current population genetics. There is too much emphasis on elegant mathematics, and not enough concern for the real values of the critical parameters -in particular, in models of mutation spread and fixation, or in models of optimal mutation rates. Recombination, a crucial genetic mechanism, is misrepresented in the models. Features that looked anecdotal, such as recombination between sister chromatids and germ-line mutations are perhaps central to the mechanisms of evolution in higher organisms. My proposals on mutation strategies [34]–[36]—see also Amos [62]—lead to rather precise insights on compensatory mutations or polymorphism propagation, yet they are largely ignored by population geneticists.

With respect to bad wheels, it seems that the reproaches are mainly addressed to mechanisms that use phenotypic variability, which may or may not be special instances of Baldwin's principle. I believe that Baldwin's principle is correct, although it now requires a formal validation by population genetics. I leave it to the proponents of “smart evolutionary devices” to state whether their proposals remain within the boundaries of Baldwin's principle, or push the cursor away from Darwin and Baldwin, and closer to Lamarck?
